# A case-based approach for teaching professionalism to residents with online discussions

**Published:** 2016-01

**Authors:** MARK T. NADEAU, JAMES TYSINGER, MARCY WIEMERS

**Affiliations:** Department of Family and Community Medicine, University of Texas Health Science Center, San Antonio (UTHSCSA), USA

**Keywords:** Milestones, Professionalism, Residency, e-learning

## Abstract

**Introduction:**

Programs must demonstrate that their residents are taught and assessed in professionalism. Most programs struggle with finding viable ways to teach and assess this critical competency. UTHSCSA Family and Community Medicine Residency developed an innovative option for interactive learning and assessment of residents in this competency which would be transferrable to other programs and specialties.

**Methods:**

The innovative approach uses an asynchronous online format on Blackboard. Threaded discussions on Blackboard require thoughtful reflective writing after case assessment and critical evaluation of other resident posts. Participation, content and progress of all resident postings are monitored by administrative staff and faculty. Faculty can further engage the residents at any point to deepen the discussion and learning.

**Results:**

100% of all senior residents attained the required learning objectives. All were actively engaged in the assignments. Six cases have been developed using a Learning Matrix to demonstrate evaluation areas from the specialty specific competencies. Written feedback from residents verified the validity of case content in context of their current clinical practice. Postings by residents have provided value and insight for the faculty to access the professional development of our Family Medicine residents.  The Clinical Competency Committee evaluates all third year residents using this information specific to the professionalism milestones. By using an asynchronous online approach to case discussion, all residents are involved with all aspects of this curriculum.

**Conclusions:**

More specific measurable learning outcomes are possible using this approach. Resident participation and engagement is easier to track and monitor than a lecture-based format and easier to capture valuable data than relying on evaluation feedback. Our Annual Review process will identify areas for improvement in the existing cases and help create supplemental cases based on a needs assessment by the faculty.

## Introduction

The Accreditation Council for Graduate Medical Education (ACGME) implemented the six competencies (Patient Care, Medical Knowledge, Professionalism, Interpersonal and Communication Skills, Systems-Based Practice, and Practice-Based Learning and Improvement) in 1999 as the centerpiece of its Outcomes Project. The ACGME introduced the Next Accreditation System and introduced the Milestones in 2013, which required residencies to develop interactive learning tools to expand education in all competencies. Historically, the teaching and assessing of some competencies (e.g., Professionalism and Practice-Based Learning and Improvement) have received less attention than Medical Knowledge and Patient Care.  Teaching professionalism provides an opportunity to address issues that create dissonance in health care settings.  While there is flexibility in teaching professionalism principles, programs must incorporate strategies to teach these traits which are essential to the development of a modern professional physician (1). The ACGME guidelines clearly state that residents must demonstrate an understanding of professionalism, so reading and other passive exercises by themselves are not adequate (2). Since all residents must be involved in these learning exercises, an asynchronous approach has been very effective.

Dramatic changes in graduate medical education, advances in technology, and expectations of residents are persuading medical educators to develop innovative teaching methods (3). Online learning including online quizzes and self-directed learning modules are used to teach residents in other specialties. Case-based discussions have been used to teach concepts and skills and have been identified as a best practice to teaching professionalism (4).Online learning in non-medical areas has also grown rapidly. According to Empson, Blackboard, Inc. was reported in 2014 to have 20 million users in over 20,000 organizations for its online learning products (5). Many online courses use threaded discussions with learners to stimulate application of content, yet such discussions have not gained widespread use in residency education.

This article describes the development of an innovative approach to teaching professionalism to residents using case-based online threaded discussions. It is based on a needs assessment that demonstrated that several professionalism topics were not being taught adequately. Cases were written by the faculty members based on learning needs identified by the faculty members who were supervising residents in the clinical setting.

Training in professionalism is essential to graduate medical education.  Since many programs lack a comprehensive curriculum in this area, program directors across specialties are trying innovative solutions to close this gap (6). Our residency faculty identified a need to improve professionalism instruction for residents. The new ACGME Milestones offered guidance to help programs understand the scope of the challenge. With the Milestones, we have specific attributes to teach and assess for every resident.

Previous approaches to this subject area failed to meet specific learners’ needs. Our didactics offered limited opportunity for residents to actively engage with the material in small group discussion and lecture format over professionalism issues that present in practice. Lectures work well for content that can be assessed with recognition, but do not facilitate comprehension and application. Learners need “cues” that trigger critical thinking and engage them, even if working autonomously (7).

Blackboard has been used successfully for asynchronous discussion for learners in the health professions, and for ethics discussion (8-10). At the time our program was developing its first professionalism case, our institution was implementing training related to our ethical duty to disclose errors to patients. The residents received some training in lecture format in Grand Rounds, but we felt a need to reinforce the concept.

The objective was to develop a systematic approach that ensured material dissemination while assessing residents’ understanding and application. The plan was to facilitate an engaging case discussion to enhance professional growth and have better data to evaluate our residents. We recognized the future potential to use these experiences to provide a summary evaluation for a given professional competency of a resident’s performance for our Clinical Competency Committee. Previously, much of our available assessment of progress in professionalism was based on general observation and evaluation by core faculty members. 

## Methods

We implemented an asynchronous, case-based online learning process for professionalism at a university-based family medicine residency. The nature of online cases requires residents to reflect on the content and share these deliberations in writing with their colleagues and faculty. The attempt was to increase successful engagement of residents in reflective writing. This approach additionally gives residents the opportunity to receive feedback and improve their written communication skills.

Faculty members identified specific gaps in residents’ knowledge of professionalism, based on observed behaviors in the clinical setting. These issues and behaviors were discussed by our faculty in the context of our curriculum and the specialty specific milestones. Our residency leadership decided that a synchronous online learning would be the best approach to ensure that all residents were taught and evaluated in these areas. Ethical concepts that were not being taught or evaluated were identified and organized using a matrix developed based on the milestones.

A web-based approach seemed attractive for four reasons: 1) Due to different clinical schedules, all residents can rarely attend every lecture and workshop. 2)  Online education facilitates engagement by all learners. Permanent postings on the discussion board permit faculty to ensure that each resident has participated in the case discussion. 3) Online learning allows residents to complete reflective writing exercises which permit assessment. 4) Case-based discussion enhances engagement and critical thinking which is more effective compared to lecture format producing only passive learning and limited participation (1, 3).

Blackboard has a very flexible and intuitive format. Our residency is organized on Blackboard in a fashion similar to an online course. Our residents access Blackboard regularly to retrieve and submit rotation-specific teaching assignments. Most residents used the online discussion tool for previous courses, but we had not extensively used this function for residency teaching.

Unlike typical lectures or workshops, the online approach allowed us to plan the learning activity to ensure 100% participation by the residents. The case was posted with enough time allowed for responses that other resident responsibilities did not interfere with participation. Additionally, the approach allowed residents to complete focused reading before posting a response, unlike a live discussion in a classroom. One of the assignments required the residents to search for supporting information before responding. 

Case development focused on specific gaps identified in an assessment of our curriculum.  The availability of Blackboard as a learning platform was seen as an opportunity to introduce online learning supported by threaded discussions. E-learning technology has been used throughout the world successfully to educate students in many settings, including healthcare education for many years (11). Small group, problem-based learning has been used successfully to teach medical ethics (12, 13). Our goal was to create a positive small group atmosphere online. Each residency class has 12-13 residents who work together in multiple clinical settings. We believed that the pre-existing familiarity with others in the group would create an immediate rapport to enhance the quality of the discussions and to ensure a supportive environment.

Case 1 was developed in 2008 to enhance our professionalism curriculum and test this online approach. Since we had no precedent for using online discussion within the residency, the format was based on previous faculty experiences with online discussion. The first case was well received. Most residents responded on time with thoughtful comments. A few residents required encouragement to finish the exercise. Our office staff monitored the discussion board and, as the deadline approached, informed the program director about resident progress. Based on this success, we developed more cases to enhance other curriculum areas.

Case 2 was developed as a result of specific educational needs identified in our practice. An adult patient with special needs presented with questions about birth control. One of our faculty was surprised by the lack of understanding a senior resident had about the patient’s autonomy. Questions from other residents led faculty to conclude our residents’ knowledge was insufficient, and a review of our curriculum found that the issue of patient autonomy had inadequately been addressed.

Case 3 was developed as the result of the program director’s concern about potential disclosure of protected health information (PHI) in online discussions. At the time our residency and department had a non-specific policy on use of the internet and resident participation on social media. A PubMed search revealed a developing body of knowledge that lacked consensus. A Google search identified a few forward thinking institutions with established policies. Since the concern and the institutional risk were substantial, we developed a policy for the residency. This case was developed to generate a discussion about the use and importance of censorship with social media.

When the draft versions of the Family Medicine Milestones became available before the July 1, 2014 implementation date, we saw the opportunity to create new cases based on the specific expectations for the residents that are outlined in the Milestones. These explicit educational requirements led to a faculty discussion at our Clinical Competency Committee (CCC) meeting about how we were teaching and assessing these specific areas. Based on this discussion we developed an educational matrix related to the competencies (see Table 1).  We made an assessment of which level of each sub-competency the case addressed and have summarized that in the table. We used the matrix as a framework to consider which topic areas might be best for future cases or as additions to the existing cases.

Our cases tend to be straightforward, easy to read, and brief. We typically have several questions to stimulate thought and comment from the residents (Example 1).

Sample Case

Case history: A 25 yo female patient from a group home with an intellectual disability presents to your clinic with a request for birth control pills. Her mother attends the visit with the patient and advocates forcefully for referral for a sterilization procedure. The patient currently is able to perform basic activities of daily life and has been working at a minimum wage job as custodial staff member at the local YMCA. During your history, the patient states that she has a boyfriend and they have been sexually active.

1. Should you respect the wishes of the mother and refer the patient for a tubal ligation?
2. Does the patient have the autonomy to consent to sex? Is this a right?
3. Does the patient have the autonomy to consent to birth control pills?
4. What would be your concerns about BCPs vs. other methods of contraception?
5. What resources are available to help you inform your patient?
6. If you were the medical director for this patient’s group home, would you consider any changes to your preventive health program for the residents of the home?

Three additional cases were developed and mapped to the matrix to fill some of the gaps. 

**Table 1:** Case development matrix

**Matrix**		**Case number**					
**Competency**	**Sub-competency**	1	2	3	4	5	6
**Patient care**	1						
	2						
	3		2,3*		2		
	4						
	5						
**Medical knowledge**	1						
	2		2,3,4				2
**Systems-based practice**	1		2,3				
	2	2,3				2	2,3
	3		3			3	
	4		3			3	
**Practice-based learning and improvement**	1						
	2						
	3	2	2				2
**Professionalism**	1			2,3,4		2	
	2			3		3	
	3		2,3,4		2,3		
	4						
**Interpersonal and communication skills**	1		2			2	
	2		2,3				2
	3					2	
	4			2,4		3	

*Numbers in table refer to the level within each sub-competency

Table 2 displays the title of each case. Future cases will be developed based on the gaps highlighted by the matrix.

**Table 2:** Case titles

**Rows**	**Case titles**
**1**	Revealing medical errors to your patients
**2**	Autonomy in a learning disabled patient
**3**	Professionalism concerns when using social media
**4**	Healthcare disparities
**5**	Managing a disruptive patient in the practice
**6**	Patient safety: A near miss

## Results

Teaching professionalism requires stating clear expectations and assessing learners according to these expectations (1), and it competes with many other areas in the curriculum. The online format has allowed us to fit this training into the limited work hours currently approved by the ACGME. There is broad agreement in the graduate medical education community that programs should have latitude to introduce some innovative methods to achieve the desired outcomes of residency training (14). By introducing this innovative method, we increased resident interest and participation. Since much of resident learning is case-based, using this approach has created a familiar atmosphere for our residents to discuss complex issues.

Of the thirteen residents eligible for participation, 100% completed all assignments and attained the required learning objectives. The length and depth of the online discussion indicated that these senior residents were actively engaged in the assignments. Six cases have been developed using a learning matrix to demonstrate evaluation areas from the specialty specific competencies. Written feedback from residents verified the validity of case content in context of their current clinical practice. The format fostered collaboration in learning by allowing residents the opportunity to review and critique the comments of their classmates asynchronously, and the residents found this to be an interesting and fun way to learn. 

A quote from one of the participating residents about the cases:“THEY ARE AN AWESOME ADDITION to our learning!! Fantastic topics, both VERY relevant, and I am intrigued by my colleagues’ responses. I hope we continue these open discussions. I truly enjoyed working on this!”

The Clinical Competency Committee (CCC) is required by the ACGME to evaluate resident academic progress. Our CCC meeting discussions included much consideration about how we knew we were teaching all necessary levels within the competencies.  The learning matrix has allowed us to think specifically about how we know that residents have achieved a specific level on the Milestones requirements. Reviewing each bullet point in each milestone against each case, we made a determination as to whether we felt the case addressed that level of competency. This gave us data to evaluate the progress of each resident’s professionalism. The matrix displays our view of the levels which each case could potentially address in the case discussion.

For example, we felt that the sample case, Case 2, addressed the bullet points in Table 3 in the Professionalism 3 Milestone (Demonstrates humanism and cultural proficiency).

**Table 3:** Professionalism 3 (Demonstrates humanism and cultural proficiency)

**Level 2**	Displays a consistent attitude and behavior that conveys acceptance of diverse individuals and groups, including diversity in gender, age, culture, race, religion, disabilities, sexual orientation, and gender identity
**Level 3**	Incorporates patients beliefs, values, and cultural practices in patient care plans
**Level 4**	Anticipates and develops a shared understanding of needs and desires with patients and families; works in partnership to meet these needs

Competencies and levels that do not appear on the matrix will require further attention. Many of these gaps are better taught in other ways within the curriculum, or additional cases may be developed in the future to address levels not appearing on the matrix.

The online cases have been formative exercises for the residents. The program director responds online to the residents’ responses. Feedback from other members of the residency class is also informative. Additional references are posted, deepening the discussion and giving the residents an opportunity to improve their information search skills. Literature searching, written communication, and case-based learning are important lifelong developmental skills for physicians. This broad set of learning skills will help our graduates acquire new knowledge and translate it into practice for their future success (4).

As a faculty, we believe there is potentially some valuable information for assessment of resident progress in Professionalism Milestones. We are developing a process to improve the quality of evaluation related to these online discussions. Our Clinical Competency Committee (CCC) plans to use the results of this information to help determine if residents have attained the desired level of mastery of some milestones. Table 4 lists several examples where resident responses to the cases matched previous impressions that the faculty had about the resident’s prior performance. This consistency helps bolster our impression that the case responses contain information which can be used to better evaluate resident development. This would be very valuable to the CCC for our residency and may be an appropriate tool for other programs and specialties. There are relatively few studies about e-learning development, implementation and needs assessment. E-learning and teaching is relatively new to many residency faculty(15). We think that practical tools designed to teach skills that are outlined in the milestones will be useful to medical education on many different levels. Table 3 lists additional observations that our Clinical Competency Committee found helpful for resident evaluation.

The following issues were identified as limitations of this approach:

(1) Faculty time commitment: Besides the time for developing cases, the faculty member must organize the discussion to facilitate resident involvement and initiate follow-up questions to enhance residents’ critical thinking. While it does not seem to be essential, the faculty member facilitating the online discussion may respond to all (or nearly all) of the responses to encourage residents to comment on additional aspects of the case. All supporting documents and articles must also be reviewed.
(2) Administrative time requirements: Although these activities tend to occur in bursts, it requires an administrative person who is able to track resident completion of tasks and encourage all participants to meet deadlines over a period of 3-6 months.
(3) Access to online learning software: Blackboard or a similar product to record the discussions requires a commitment of resources that may be difficult for some programs.
(4) Milestone interpretation: Faculty members elsewhere may come to different conclusions about whether a learner has met a milestone  Despite this limitation due to milestone level interpretation, this approach gave us an logical starting point to view whether we were addressing all competencies at all levels

**Table 4:** Correlating case performance with known resident attributes

**Case performance**	**Resident attribute**
Missing case deadline	Prior punctual concern
Minimalist participation	Prior overall professionalism concerns
Incomprehensible response	English as a second language
Recalling prior events in conflict with current postings	Insight into professional development

## Discussion

Using this creative approach, we now cover a number of critical professionalism areas that were previously not addressed in our curriculum. This article’s goals are to describe how our faculty identified a major educational gap, how the innovation was implemented in our residency, and how the innovation met the criteria for transferability to other programs and specialties (16). The instructional innovation has been sustainable in our setting. Starting with one case in 2008, our program has seven years of experience using the threaded discussion to teach professionalism. While the approach can be generalized to other residencies in other specialties, the cases may require revision if used in other training programs. New cases may be written based on a local needs assessment. Blackboard and other readily available software platforms can be configured to encourage residents’ asynchronous learning. Our next phase in this process is to measure specific learning outcomes from the cases. Formal evaluation and feedback from the residents about the cases will provide data to improve their perceived experience from this learning activity (17).

## Conclusion

The matrix will allow us to re-evaluate our initial assessments in Table 1 as to which sub-competencies are addressed and whether they are addressed adequately. Future cycles of improvement in our matrix will be documented in our Annual Program Review of Effectiveness, which is the essential place to record ongoing efforts at program improvement.
